# Effect of Menin Deletion in Early Osteoblast Lineage on the Mineralization of an In Vitro 3D Osteoid-like Dense Collagen Gel Matrix

**DOI:** 10.3390/biomimetics7030101

**Published:** 2022-07-22

**Authors:** Ildi Troka, Gabriele Griffanti, Lucie Canaff, Geoffrey N. Hendy, David Goltzman, Showan N. Nazhat

**Affiliations:** 1 Faculty of Dental Medicine and Oral Health Sciences, McGill University, Montreal, QC H3A 1G1, Canada; ildi.troka@mail.mcgill.ca; 2 Department of Mining and Materials Engineering, McGill University, Montreal, QC H3A 0C5, Canada; gabriele.griffanti@mail.mcgill.ca; 3 Department of Medicine, McGill University and McGill University Health Centre, Montreal, QC H4A 3J1, Canada; lucie.canaff@gmail.com (L.C.); david.goltzman@mcgill.ca (D.G.)

**Keywords:** menin, plastic compression, dense collagen, mineralization, osteoblastic differentiation

## Abstract

Bone has a complex microenvironment formed by an extracellular matrix (ECM) composed mainly of mineralized type I collagen fibres. Bone ECM regulates signaling pathways important in the differentiation of osteoblast-lineage cells, necessary for bone mineralization and in preserving tissue architecture. Compared to conventional 2D cell cultures, 3D in vitro models may better mimic bone ECM and provide an environment to support osteoblastic differentiation. In this study, a biomimetic 3D osteoid-like dense collagen gel model was used to investigate the role of the nuclear protein menin plays in osteoblastic differentiation and matrix mineralization. Previous in vitro and in vivo studies have shown that when expressed at later stages of osteoblastic differentiation, menin modulates osteoblastogenesis and regulates bone mass in adult mice. To investigate the role of menin when expressed at earlier stages of the osteoblastic lineage, conditional knockout mice in which the *Men1* gene is specifically deleted early (i.e., at the level of the pluripotent mesenchymal stem cell lineage), where generated and primary calvarial osteoblasts were cultured in plastically compressed dense collagen gels for 21 days. The proliferation, morphology and differentiation of isolated seeded primary calvarial osteoblasts from knockout (*Prx1-Cre; Men1f/f*) mice were compared to those isolated from wild-type (*Men1f/f*) mice. Primary calvarial osteoblasts from knockout and wild-type mice did not show differences in terms of proliferation. However, in comparison to wild-type cells, primary osteoblast cells derived from knockout mice demonstrated deficient mineralization capabilities and an altered gene expression profile when cultured in 3D dense collagen gels. In summary, these findings indicate that when expressed at earlier stages of osteoblast differentiation, menin is important in maintaining matrix mineralization in 3D dense collagen gel matrices, in vitro.

## 1. Introduction

Collagen is the most abundant protein in the human body and the main component of connective tissues such as bones [1]. In particular, the extracellular matrix (ECM) of bone is rich in type I collagen, which represents a preformed unmineralized matrix, called the osteoid and serves as the native scaffolding material for bone formation [2]. The ECM is secreted by osteoblasts, which then self-assembles either in the extra-cellular space or inside intracellular vesicles followed by the secretion of a package of aligned fibrils [3]. The calcium and phosphate ion-containing vesicles are then secreted into the ECM in the form of amorphous minerals. Once secreted, the amorphous minerals crystallize within and between collagen fibrils resulting in the mineralization of the osteoid [3].

For this reason, type I collagen-based hydrogel scaffolds are of significant interest as biomaterials and can be used to recreate tissue-like constructs such as the osteoid [1]. However, due to the fact of their highly hydrated nature, the mechanical and structural properties of traditional collagen hydrogels are far from those of the native osteoid [1]. Compaction techniques, such as the plastic compression of collagen gel [4], has been used to overcome this limitation, thus resulting in a dense gel matrix characterized by a well-packed network of collagen fibrils resembling the native structure of the bone ECM. Dense, plastic compressed [4] collagen gels have been extensively used as in vitro 3D models, attributable to their capacity to better mimic the physiological bone microenvironment, supporting both cellular differentiation and cell-mediated matrix mineralization process [4,5,6,7,8,9] and is reviewed in [1]. For this reason, dense collagen gels represent a powerful 3D model to study not only physiological but also pathological processes involved in bone development.

Menin is a protein product of the multiple endocrine neoplasia type-1 (*MEN1*) gene, widely expressed in many tissues of the body [10,11]. The expression of menin begins early in the development and continues postnatally [12]. More than 500 independent mutations of *MEN1* leading to the hereditary autosomal dominant MEN1 disorder have been identified [13]. It has also been demonstrated that menin acts as a molecular rheostat regulating the balance of mesenchymal stem (MSC) cell commitment to both osteogenic and myogenic lineages [14].

Germline homozygous inactivation of the *MEN1* gene is predicted to be lethal in patients. However, individuals with heterozygous *MEN1* gene mutation often exhibit parathyroid tumors and have high parathyroid hormone (PTH) levels due to the fact of overproduction and secretion from the parathyroid cells of the parathyroid gland [13,15,16]. As a result, it is difficult to evaluate the direct effects of heterozygous *MEN1* mutations on bone in MEN1 patients, as it is unclear if such effects are due to the direct consequence of the *MEN1* gene disruption within bone cells or to high PTH levels, which can also affect bone metabolism, or both. For this reason, analysis of the function of menin in bone homeostasis has mainly been performed either two-dimensionally, in vitro or in vivo on animal models. These investigations have provided more information regarding the role that menin plays downstream of signaling pathways important for osteoblastogenesis. Menin is known to be critical for the proper functioning of mature osteoblasts and maintenance of bone mass in adult mice [17]. In particular, when *Men1* gene is specifically deleted in mature osteoblasts using *OC-Cre, Men1f/f* transgenic mice, menin regulates osteoblastic differentiation and mineralization and controls apoptosis of mature osteoblasts [18].

In the current study, the role of menin, when expressed at earlier stages of osteoblastic differentiation, was investigated in a biomimetic osteoid-like 3D dense collagen gel in vitro. It was demonstrated that the early expression of menin in the osteoblastic lineage plays a crucial role in the cell-mediated matrix mineralization process of this biomimetic osteoid-like model.

## 2. Materials and Methods

### 2.1. Mice Breeding

*Men1^flox/flox^* mice that possessed loxP sites flanking exons 3–8 of the *Men1* gene [19] and *Pair-related homeobox gene 1-Cre* transgenic mice (*Prx1-Cre^TG/+^*) were both obtained from the Jackson Laboratory (Bar Harbor, ME, USA). For the generation of conditional knockout mice in which menin was specifically deleted at the level of the MSC, *Men1^flox/flox^* mice (129S(FVB)-*Men1^tm1.2Ctr^*^e^) that possessed loxP sites flanking exons 3–8 of the *Men1* gene were first crossed with *Prx1-Cre^TG/+^* mice (B6.Cg-Tg(Prrx1-cre)1Cjt/J) which express the Cre recombinase. The resulting *Prx1-Cre^TG/+^, Men1^+/flox^* mice were then crossed with *Men1^flox/flox^* mice to generate *Prx1-Cre^TG/+^/Men1^flox/flox^* mice. These were then crossed with *Men1^flox/flox^* to obtain litters where all mice had a floxed *Men1* gene, but where only half would express the Cre recombinase transgene. To maintain the mice colony *Prx1-Cre^TG/+^, Men1^flox/flox^* mice were crossed with *Men1^flox/flox^* mice. The control littermate mice were designated as *Men1f/f* and knockout mice as *Prx1-Cre, Men1f/f*. These mice were on a mixed FVB/C57BL/6J background.

Mice were maintained in a pathogen-free standard animal facility, and experimental procedures were performed following an animal use protocol approved by the animal Care and Use Committee of McGill University. This protocol also conformed to the ethical guidelines of the Canadian Council on Animal Care. Mice colonies were maintained on a 12 h alternating light/dark cycle at 22–26 °C and fed a rodent 18% protein chow diet (Envigo (TEKLAD global), Madison, WI, USA; Product# T.2918.15) containing 1.0% calcium, 0.7% phosphorus and 1.5 IU vitamin D_3_/g.

### 2.2. Genotyping

Genomic DNA was obtained and isolated from *Men1f/f* and *Prx1-Cre, Men1f/f* mice tail snips using the Quanta bioscience DNA extraction kit. The 2x green PCR Master-mix (ZmTech Scientifique, QC, Canada) was used for polymerase chain reactions (PCRs). Three different primers were employed for detecting the presence of either wild-type or floxed *Men1* alleles. Primer A (5′—CCCACATCCAGTCCCTCTTCAGCT—3′) was specific to exon 2 of the *Men1* gene. Primer B (5′—CCCTCTGGCTATTCAATGGCAGGG—3′) was specific to the wild-type sequence in intron 2 that was deleted in the cloning of the loxP sequence. Primer C (5′—CGGAGAAAGAGGTAATGAAATGGC—3′) was specific for the inserted loxP sequence. PCRs with primers A and B generated a 300 bp amplicon, while PCR with primers A and C gave a 236 bp amplicon. *Men1^flox/+^* mice were identified as the ones having both the 300 bp and 236 bp amplicons, while *Men1^flox/flox^* mice would only show the 236 bp amplicon on an agarose gel. The 2x green PCR Master-mix (ZmTech Scientifique, QC, Canada) was also used for detecting the presence of the Cre recombinase. The two primers used for detecting the Cre transgene were forward 5′—CTAGGCCACAGAATTGAAAGATCT—3′ and reverse 5′—GTAGGTGGAAATTCTAGCATCATCC—3′. The amplicon using these two primers corresponded to the internal positive control sequence that was put next to the Cre-recombinase transgene sequence in the transgenic mice provided by the Jackson Laboratory. *Prx1-Cre^TG/+^* mice were identified as those possessing a 100 bp amplicon, whereas *Prx1-Cre^+/+^* mice, not expressing the Cre transgene, would show no PCR product when using these two primers.

For genotyping, the PCR conditions, using the T100TM Thermal Cycler (Bio-Rad, Hercules, CA, USA), were as follows: 32 cycles of denaturation at 95 °C for 30 s, annealing at 60 °C for 30 s and elongation at 72 °C for 1 min. The PCR products were separated by gel electrophoresis on 2% agarose gel and were visualized using the Fluo-DNA/RNA gel staining solution (ZmTech Scientifique, Montréal, QC, Canada) with ultraviolet (UV) light using an electronic ultraviolet transilluminator (Alpha Innotech, San Jose, CA, USA).

### 2.3. Primary Calvarial Osteoblast Isolation

Individual 5–6 month old knockout mice and their control wild-type littermates were euthanized, and their calvarias were isolated as previously described [20]. Each calvaria was washed for 2 s in 70% ethanol and then rinsed with 1 mL of PBS 1X. The calvaria were then dissected under sterile conditions in PBS 1X, making sure to remove sutures and soft tissues with a sterile scalpel. Each individual calvaria was chopped into small fragments of approximately 1–2 mm^2^ and then transferred into a sterile conical flask in order to begin a series of enzymatic digests. One millilitre of freshly prepared solution I (10 mL α-MEM (Gibco, Life Technologies, Grand Island, NY, USA), 125 µL 0.25% trypsin (Wisent Inc.) + 5 µL collagenase P (Roche Diagnostics, Indianapolis, IN, USA) (100 mg/mL)) was then added in each conical and then incubated at 37 °C for 15–20 min while shaking. Solution I was then discarded and replaced with 1 mL of solution II (10 mL α-MEM (Gibco, Life Technologies, USA), 125 µL 0.25% trypsin (Wisent Inc.) + 10 µL collagenase P (Roche Diagnostics, Indianapolis, USA) (100 mg/mL)) at 37 °C for 30 min while shaking. Lastly, solution II was aspirated and replaced with 1 mL of solution III (10 mL α-MEM (Gibco, Life Technologies, Grand Island, NY, USA), 125 µL 0.25% trypsin (Wisent Inc., St Bruno, QC, Canada) + 100 µL collagenase II (Roche Diagnostics, Indianapolis, USA) (100 mg/mL)) at 37 °C for 60 min while shaking. For this last digestion, the tubes were shaken vigorously every 10 min. Solution III was then aspirated, and the fragments were washed with a solution of α-MEM (Gibco, Life technologies, USA) supplemented with 10% fetal bovine serum (FBS) (Wisent Inc.) and 1% penicillin–streptomycin (Wisent Inc.). Calvaria fragments were then plated on a 10 cm petri-dish and cultured for 5–7 days, until confluent, in α-MEM (Gibco, Life Technologies, USA) supplemented with 10% FBS (Wisent Inc.) and 1% penicillin–streptomycin (Wisent Inc.). After reaching adequate confluence, the cells were trypsinized with 0.25% trypsin/EDTA (Wisent Inc.) in order to detach the cells that migrated out of the calvaria fragments. For subsequent experiments, the bone cells were plated in differentiation medium containing 50 µg/mL L-ascorbic acid (Sigma-Aldrich, St. Louis, MO, USA) and 10 mM β-glycerophosphate (Sigma-Aldrich) at a density of 5000 cells/cm^2^, with the cell medium being freshly renewed 3 times per week.

### 2.4. 3D Dense Collagen Gel Fabrication

Plastically compressed collagen gels were used as 3D scaffolds and were prepared as previously described [4]. Briefly, 4 mL of 2 mg/mL sterile rat-tail-derived type I collagen (First Link Ltd., UK) was mixed with 1 mL of 10X DMEM (Sigma-Aldrich), followed by drop-wise neutralization with 5 M NaOH. After neutralization, primary calvarial osteoblasts from 6 month old *Prx1-Cre, Men1f/f* and *Men1f/f* mice were homogenously seeded into the collagen solution at a cell density of 60,000 cells/mL. The solution was then transferred to a 48-well plate (1.5 mL for each well) followed by 30 min incubation in a 5% CO_2_ incubator at 37 °C. After gelling, highly hydrated hydrogels were plastically compressed through application of 1 kPa compressive stress, thus producing dense collagen scaffolds of approximately 10% collagen fibrillar density (collagen content). Each gel was then transferred into a 6-well plate and cultured with α-MEM medium (Gibco, Life technologies, USA) supplemented with 10% FBS (Wisent Inc.) and 1% penicillin/streptomycin (Wisent Inc.). After a 48 h recovery time, α-MEM medium was replaced with osteogenic differentiation medium consisting of α-MEM medium supplemented with 10% FBS (Wisent Inc.), 1% penicillin/streptomycin (Wisent Inc.), 50 μg/mL ascorbic acid (Sigma, Oakville, ON, Canada) and 10 mM β-glycerolphosphate (Sigma, Oakville, ON, Canada). The cell-seeded collagen gels were maintained in culture under osteogenic condition for up to 21 days in a 5% CO_2_ incubator at 37 °C. The cell media was renewed 3 times a week.

### 2.5. Live/Dead Assay

Confocal laser scanning microscopy (Carl Zeiss LSM510, Toronto, ON, Canada) was used to image the viability and morphology of primary calvarial osteoblasts from 6 month old *Prx1-Cre, Men1f/f* and *Men1f/f* mice seeded in plastically compressed dense collagen gels at days 1 and 21 in culture medium. Seeded cells were stained with 2 μM calcein-AM and 4 μM ethidium homodimer-1 (Live/Dead assay, Life Technologies, Canada) solution (i.e., in culture media) and incubated at 37 °C for 15 min prior to viewing. Excitation by argon laser (488 nm) allowed for detection of fluorescence detection as green for live calcium-laden cells, while excitation by HeNe laser (543 nm) allowed for detection of fluorescence detection as red binding exposed (compromised or dead cells) nuclear content.

### 2.6. AlamarBlue^®^ Assay

Cell metabolic activity among the dense collagen scaffolds (*n* = 3, biological replicates) was assessed using the alamarBlue^®^ assay (ThermoFisher Scientific, Waltham, MA, USA) at days 2, 10, 15 and 21 of osteogenic differentiation. Seeded gels were stained in osteogenic medium with 10% alamarBlue^®^ reagent and incubated under darkness in 5% CO^2^ and 37 °C for 3 h. After the incubation period, the fluorescence was read using excitation and emission wavelengths of 530 and 610 nm with a microplate reader (SynergyTM H4 Hybrid Multi-Mode Microplate Reader, BioTek, Shoreline, WA, USA). Data were normalized against the fluorescent intensity at day 1.

### 2.7. RNA Isolation, RT-qPCR

For gene expression analyses of primary cells seeded within 3D scaffolds, quantitative reverse transcription PCR (RT-qPCR) was performed. Total RNA was extracted using the standard TRIzol reagent (Invitrogen, Frederick, MD, USA) method as recommended by the manufacturer. For each cell-seeded gel, 250 ng of total RNA was employed for the synthesis of single-stranded cDNA using the high-capacity cDNA reverse transcription kit (Applied Biosystems, California, USA). RT-qPCR, using the Power SYBR Green PCR Master Mix (ThermoFisher Scientific, USA), was performed to estimate the abundance of specific mRNAs relative to GAPDH mRNA. The following primers were used: *Men1* forward 5′—GTGGCCGACCTATCCATCATT—3′, *Men1* reverse 5′—GGCCCGGTCCTTGAAGTAG—3′; *Runx2* forward 5′—GCTCACGTCGCTCATCTTG—3′, *Runx2* reverse 5′—TATGGCGTCAAACAGCCTCT—3′; *Ocn* forward 5′—CAGACAAGTCCCACACAGCA—3′, *Ocn* reverse 5′—CTTGGCATCTGTGAGGTCAG—3′; *Alp* forward 5′—GGGAGATGGTATGGGCGTCT—3′, *Alp* reverse 5—AGGGCCACAAAGGGGAATTT—3′; *Spp1* forward 5′—TGGCTATAGGATCTGGGTGC—3′, *Spp1* reverse 5′—ATTTGCTTTTGCCTGTTTGG—3′; *Col1α1* forward—CTGGCGGTTCAGGTCCAAT—3′, *Col1α1* reverse 5′—TTCCAGGCAATCCACGAGC—3′; *Ibsp* forward 5′- GAAGAGTCACTGCCTCCCTG—3′, *Ibsp* reverse 5′- TCCATCGAAGAATCAAAGCA—3′; *Gapdh* forward 5′—CACCATCTTCCAGGAGCGAG—3′, *Gapdh* reverse 5′—CCTTCTCCATGGTGGTGAAG—3′.

The PCR conditions, using the ViiATM 7 Real-Time PCR (Applied Biosystems, Waltham, MA, USA), were as follows: 40 cycles of denaturation at 95 °C for 15 s, annealing 56–60 °C for 45 s and elongation at 72 °C for 60 s. All cDNA samples were run in triplicate, averaged and normalized to *Gapdh*, which was used as the housekeeping gene. The Ct method was used to assess changes in expression levels for each gene.

### 2.8. Scanning Electron Microscopy (SEM)

Scanning electron microscopy (SEM), at days 1 and 21 in culture, was performed on primary calvarial osteoblast-seeded dense collagen gels from 6 month old male *Prx1-Cre, Men1f/f* and *Men1f/f* mice to analyze cell-mediated mineralization processes, scaffold microstructure and cellular morphology. Gels were fixed in 4% paraformaldehyde overnight, then rinsed with deionized water for 10 min, followed by dehydration through an ethanol gradient followed by immersion in 1,1,1,3,3,3-hexamethyldisilazane (Sigma-Aldrich). Dehydrated samples were coated with Pt using a Leica Microsystems EM ACE600 sputter coater (Austria). Images were acquired with a FEI Inspect F-50 FE-SEM (FEI Company, Hilsboro, OR, USA) at 5 kV. Energy-dispersive X-ray spectroscopy (EDS) was used for chemical characterization of particles deposited in seeded gels and performed at 10 kV.

### 2.9. Attenuated Total Reflectance-Fourier Transform Infrared (ATR-FTIR) Spectroscopy

ATR-FTIR spectroscopy was used to characterize cell-mediated matrix mineralization at days 1, 15 and 21 in culture. Gels were fixed in 4% paraformaldehyde overnight, then rinsed with deionized water for 10 min and finally dehydrated through an ethanol gradient followed by immersion in 1,1,1,3,3,3-hexamethyldisilazane (Sigma-Aldrich). Spectra were collected with a Spectrum 400 (Perkin Elmer, Waltham, MA, USA) using a resolution of 2 cm^−1^, an infrared range of 4000–650 cm^−1^ and 64 scans. Spectra were then corrected with a linear baseline and normalized (absorbance of Amide I at 1643 cm^−1^ = 1.5) using Spectrum software (Perkin Elmer, Waltham, MA, USA). The mineral-to-matrix ratio (*n* = 3) was calculated by dividing the area under the phosphate (mineral) peak (900–1200 cm^−1^) by the area under the Amine I (matrix) peak (1590–1720 cm^−1^) after both peaks were background and baseline shift corrected [21]. The carbonate to phosphate ratio (*n* = 3) was calculated by dividing the area under the carbonate peak (850–890 cm^−1^) by the area under the phosphate peak [21].

### 2.10. X-ray Powder Diffraction (XRD)

XRD was used to characterize cell-mediated matrix mineralization at days 1, 15 and 21 in culture. XRD patterns of dehydrated gels (as described above) were recorded with a Bruker D8 Discover (Milton, ON, Canada) from 6 to 60 2θ degree at 40 kV and 40 mA. Three frames of 30° were recorded for 10 min and then merged during data postprocessing. Phase composition was determined by comparing the acquired diffraction pattern with peaks identified in the International Centre for Diffraction Data (ICDD) database.

### 2.11. Statistical Analysis

Statistical differences between groups were determined using the two-tailed unpaired Student’s *t*-test with the GraphPad Prism analysis software version 7.0 (GraphPad Software, Inc., San Diego, CA, USA). For all statistical tests, a *p*-value < 0.05 was considered statistically significant (* *p* < 0.05; ** *p* < 0.01; *** *p* < 0.001; **** *p* < 0.0001).

## 3. Results

**Figure 1:**
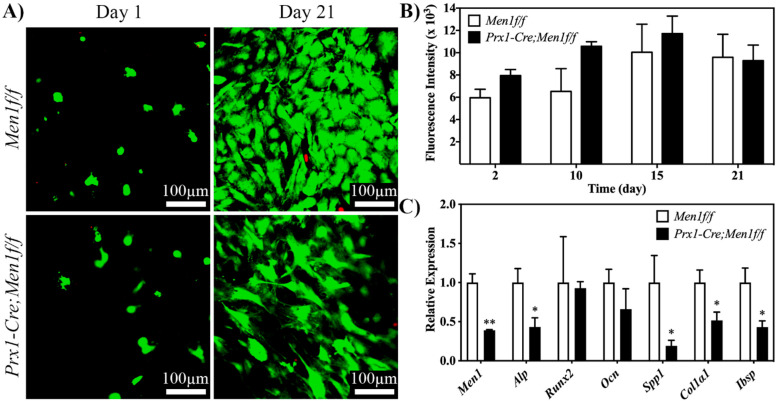
Viability, proliferation and osteogenic differentiation of calvarial osteoblasts from 6 month old *Men1f/f and Prx1-Cre*, *Men1f/f* mice seeded in 3D dense collagen gels under osteogenic medium in vitro. (**A**) Confocal images of calcein-AM and EthD-1-stained cells at days 1 and 21. No qualitative differences between the two groups were observed (*n* = 3). (**B**) Metabolic activity of seeded cells was assessed at days 2, 10, 15 and 21 of osteogenic differentiation. Seeded gels were stained in osteogenic medium with 10% alamarBlue^®^ reagent, and fluorescence was determined using a microplate reader. No significant differences between the two groups were observed (*n* = 3). (**C**) Expressions of *Men1*, *Alp*, *Runx2*, *Ocn*, *Spp1*, *Col1α1* and *Ibsp*, of seeded cells at day 21. Significantly (* *p* < 0.05 and ** *p* < 0.01) lower expressions of markers were observed in *Prx1-Cre, Men1f/f* compared to *Men1f/f* osteoblasts (*n* = 3).

The effect of the early deletion of menin in the osteoblast lineage, in terms of cell differentiation and mineral deposition, was evaluated in plastically compressed 3D dense collagen gels for up to 21 days under osteogenic medium. Confocal laser scanning microscopy images of calcein-AM and ethidium homodimer-1 stained *Men1 f/f* and *Prx1-Cre, Men1f/f* osteoblasts at days 1 and 21 demonstrated extensive cell viability and homogenous cell spreading within the gel with no qualitative differences between the two cell types (Figure 1A). The alamarBlue^®^ assay was performed to evaluate cell metabolic activity up to day 21. Both seeded cell types showed a similar trend, where their metabolic activity increased up to day 15 and then slightly decreased at day 21 (Figure 1B).

The q-PCR analysis was performed at day 21 on genes involved in the osteogenic differentiation pathway, i.e., menin (*Men1*), alkaline phosphatase (*Alp*), runt-related factor 2 (*Runx2*), osteocalcin (*Ocn*), collagen type I alpha 1 chain (*Col1α1*) and integrin-binding sialoprotein (*Ibsp*). The expression of menin was significantly lower in the menin-deleted *Prx1-Cre, Men1f/f* cells when compared to that of *Men1f/f* cells. In addition, the expression levels of *Alp*, *Spp1*, *Col1α1* and *Ibsp* were significantly lower in the menin-deleted *Prx1-Cre, Men1f/f* cells when compared with those of wild-type *Men1f/f* osteoblasts (Figure 1C).

**Figure 2:**
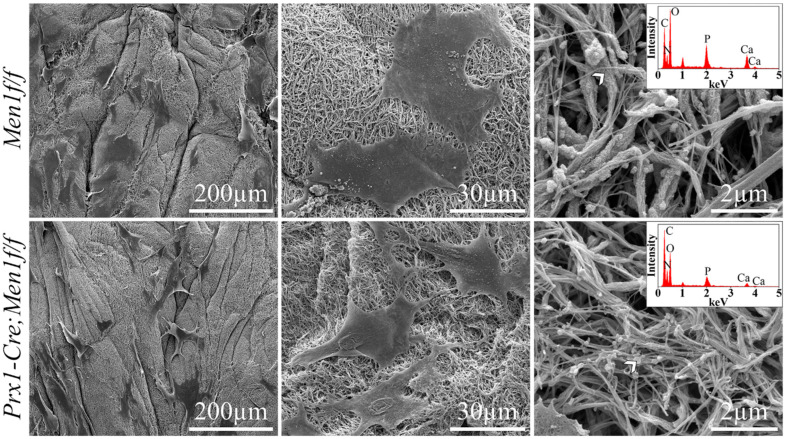
SEM micrographs at day 21 of calvarial osteoblasts from 6 month old *Men1f/f* and *Prx1-Cre*, *Men1f/f* mice 3D seeded in 3D dense collagen gels under osteogenic medium in vitro. Lower magnification SEM micrographs (left panel) depicted evenly distributed cells within the collagen gels in both groups. Higher magnification images qualitatively indicated that the morphology of *Men1f/f* cells appeared larger compared to that of *Prx1-Cre, Men1f/f* cells (middle panel). While mineralized particles were detected in both groups, mineralized collagen fibrils were only indicated in *Men1f/f* seeded gels (right panel). EDS revealed the presence of calcium and phosphorous in both groups (inset in right panel) (*n* = 3).

The SEM micrographs of cell-seeded gels at day 21 depicted a dense and randomly organized network of collagen fibrils, where both cell types appeared to be flattened and anchored through extracellular projections to the collagenous matrix (Figure 2, left and middle). While cell-mediated matrix mineralization was observed in both cell types, mineral deposition within the gels seeded with *Men1f/f* osteoblasts were characterized by both particles and collagen fibrils encrusted by mineral. On the other hand, gels seeded with *Prx1-Cre, Men1f/f* osteoblasts were only characterized by particle deposition, which qualitatively appeared to be smaller compared to those observed in gels seeded with *Men1f/f* osteoblasts (Figure 2, right). EDS analysis on the deposited particles and the layer encrusting the collagen fibrils identified both calcium and phosphorous in gels seeded with both cell types (Figure 2, right, inset). In contrast, acellular gels at day 21 displayed a dense and randomly organized network of collagen fibrils with no evidence of mineral deposition (Figure S1). Furthermore, EDS analysis identified only oxygen, nitrogen and carbon but neither calcium nor phosphorous.

**Figure 3:**
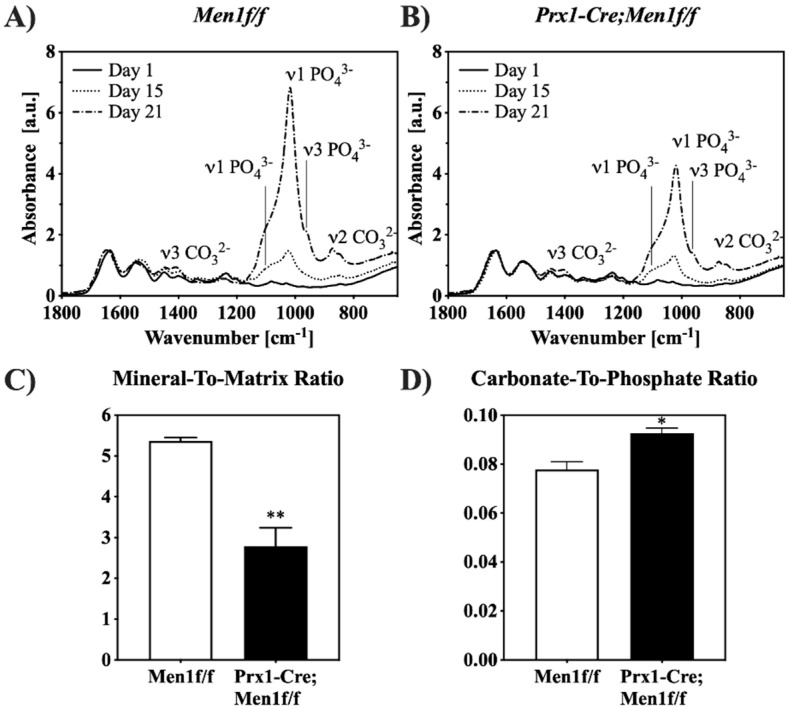
ATR-FTIR spectroscopy of cell-mediated matrix mineralization. (**A**,**B**) ATR-FTIR spectra of 3D seeded calvarial osteoblasts from 6 month old *Men1f/f and Prx1-Cre*, *Men1f/f* mice, respectively, in dense collagen gels cell-seeded collagen gels up to day 21 in osteogenic differentiation medium. Both groups exhibited a time-dependent increase in the intensity of absorption bands related to phosphate and carbonate groups (*n* = 3). (**C**) The mineral-to-matrix ratio derived from the ATR-FTIR spectra of cell-seeded collagen gels at day 21. The ratio was significantly (** *p* < 0.01) lower in *Prx1-Cre, Men1f/f* compared to *Men1f/f* seeded collagen gel (*n* = 3). (**D**) The carbonate-to-phosphate ratio derived from ATR-FTIR spectra of cell-seeded collagen gels at day 21. The ratio was significantly (* *p* < 0.05) higher in *Prx1-Cre, Men1f/f* compared to *Men1f/f* seeded collagen gels (*n* = 3).

The progression of cell-mediated mineralization was characterized through ATR-FTIR spectroscopy up to day 21 (Figure 3). In all gels, the presence of the collagen triple helix was confirmed by the absorption bands corresponding to amide I, II, and III at 1643, 1525, and 1233 cm^−1^, respectively [22]. In addition, at day 15 in culture, spectra of cell-seeded gels indicated a time dependent increase in the absorption bands related to phosphate (ν_1_ at 1070 and 1033 cm^−1^, and ν_3_ at 957 cm^−1^) and carbonate (ν_3_ at 1450 and 1420 cm^−1^, and ν_2_ at 872 cm^−1^) groups. The intensities of the absorption bands related to phosphate and carbonate groups at days 15 and 21 appeared qualitatively lower in gels seeded with *Prx1-Cre, Men1f/f* compared with those seeded with *Men1f/f* osteoblasts (Figure 3A,B). The extent of cell-mediated matrix mineralization at day 21 was semi-quantified by calculating the mineral-to-matrix ratio [23]. A significantly lower mineral content was observed in gels seeded with *Prx1-Cre, Men1f/f* compared with those seeded with *Men1f/f* osteoblasts (Figure 3C). Moreover, the carbonate-to-phosphate ratio was quantified to indicate the extent of carbonate substitution within the mineral [23]. A significantly higher ratio was observed in gels seeded with *Prx1-Cre, Men1f/f* compared with those seeded with *Men1f/f* osteoblasts, indicating a lower degree of crystal purity (Figure 3D).

**Figure 4:**
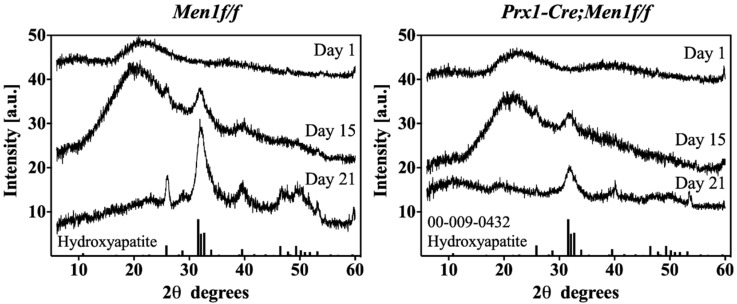
XRD diffractograms of cell-mediated matrix mineralization. Left and right diffractograms show time-dependent XRD diffractograms of 3D seeded calvarial osteoblasts from 6 month old *Men1f/f* and *Prx1-Cre*, *Men1f/f*, respectively, seeded in dense collagen gels up to day 21 in osteogenic differentiation medium. Both groups displayed a time-dependent amorphous-to-crystalline transition. *Men1f/f* seeded gels displayed higher intensity peaks relative to those of *Prx-Cre, Men1f/f* (*n* = 3).

The XRD diffractograms of all seeded gels at days 1, 15 and 21 showed a transition from an amorphous to a crystalline apatitic phase as indicated by the temporal increase in the peak at 32° of 2θ. Furthermore, the intensity of all peaks characteristic of the apatite pattern appeared lower in gels seeded with *Prx1-Cre, Men1f/f* compared with those seeded with *Men1f/f* osteoblasts, indicating a lower degree of crystallinity (Figure 4).

## 4. Discussion

This work demonstrated that the conditional knockout of menin early in the osteoblast lineage impacted the mineral deposition of primary calvarial osteoblast cells when seeded in biomimetic osteoid-like 3D dense collagen gel matrices, in vitro. Bone is an important organ with structural and supporting functions as well as having roles in regulating metabolic processes [24,25]. The processes of resorption and formation are maintained within a tightly regulated balance. Any imbalance in bone remodelling will cause metabolic bone disorders categorized through either loss (e.g., osteoporosis) or gain (e.g., osteopetrosis) in bone mass [24,25,26,27,28]. In order to design therapeutic molecules that may address the treatment of low bone mass disorders, such as osteoporosis, it is important to elucidate the functions of regulators affecting molecular pathways directly implicated in bone homeostasis.

Menin is an important mediator of osteoblastic differentiation required to maintain bone mass [19,29,30,31]. In particular, it is implicated in bone development where homozygous *Men1^-/-^* fetuses exhibited important developmental delay, neural tube defects and craniofacial defects, suggesting the potential for menin to mediate intramembranous bone ossification [32,33]. Two-dimensional in vitro studies have also reported that menin regulates TGF-β/BMP-2 signaling pathways to promote the commitment of MSCs to the osteoblast lineage and facilitate the differentiation of immature osteoblasts [29,30]. In MSCs, menin interacts with Smads 1/5 and Runx2, regulating the expression of genes downstream the signaling pathway important for osteoblast differentiation [30]. Moreover, in vivo studies have confirmed the role of menin in mice cranial and rib bone development, as well as its role in maintaining bone mass [31]. In vivo studies have shown that in mature osteoblasts menin is required to maintain bone mass by affecting bone formation and osteoblast function [19]. When expressed at early stages of osteoblast differentiation, menin has also been shown to maintain bone mass by regulating osteoclastogenesis and bone resorption in vivo [34].

In this study, it was hypothesized that a biomimetic 3D osteoid-like dense collagen gel may be used as a tool to investigate the effect of the early deletion of menin in calvaria derived osteoblastic cells, namely on their ability to mineralize matrices, in terms of quantity and mineral morphology. Therefore, the *Men1* gene was conditionally disrupted at the level of the MSC (*Prx1-Cre, Men1f/f*). To better mimic the physiological bone microenvironment and to provide a more adequate native-ECM-like environment that supports cellular differentiation and mineralization processes relevant to bone biology, cells were seeded into plastically compressed 3D dense collagen hydrogels [4,5,6,7,8]. These were chosen as in vitro tissue models to investigate the differentiation and mineralization abilities of seeded primary osteoblastic cell when cultured for 21 days in osteogenic medium. Although viability and proliferation appeared to be similar in both *Prx1-Cre, Men1f/f* and *Men1f/f* cell-seeded gels, the gene expression patterns showed differences. While the expression of genes involved in the early stages of osteoblastic differentiation such as RUNX2 did not vary, the expression of genes implicated in osteoblast-mediated mineral deposition such as Col1α1, Alp, Spp1 and Ibsp, was significantly lower in seeded *Prx1-Cre, Men1f/f* compared with *Men1f/f* calvarial osteoblasts. These results suggest that menin regulates the function of mature osteoblasts, such as mineralization, by altering the expression of genes under the control of the TGF-β/BMP signaling pathways. The decrease in osteoblast mediated mineralization was supported by SEM microscopy and EDS, which showed that dense hydrogels seeded with *Prx1-Cre, Men1f/f* cells revealed collagen fibrils covered by overall smaller calcium-phosphate mineral particles than those seeded with *Men1f/f* cells. ATR-FTIR spectroscopy and XRD diffraction indicated a lower extent of mineralization was observed in gels seeded with *Prx1-Cre, Men1f/f* compared with those seeded with *Men1f/f* calvarial osteoblasts. These suggested a different cell-mediated mineralization process carried out by osteoblasts of knockout mice compared to those of wild-type mice. Moreover, cells isolated from the knockout mice may not be able to adequately secrete the native ECM components as suggested by the lower expression of genes implicated in the osteoblast mediated mineralization process, thus affecting the quality of mature osteoblasts.

## 5. Conclusions

The results of this study indicated that the early deletion of menin in the osteoblast lineage affects their late-stage differentiation and matrix mineralization in vitro. Overall, these results confirmed that the use of a biomimetic 3D collagenous matrix may provide better insights into the cell-mediated mineral deposition processes compared to those of 2D systems [19,34,35].

## Supplementary Materials

The following supporting information can be downloaded at: https://www.mdpi.com/article/10.3390/biomimetics7030101/s1, Figure S1: Acellular dense collagen gel at day 21 in osteogenic differentiation medium. Low (left) and high (right) magnification SEM micrographs indicating no mineral deposition. EDS (inset in right panel) on collagen fibril confirmed only the presence of oxygen, nitrogen and carbon and with no calcium or phosphorous.
