# Supplementary material for: Effect of Menin Deletion in Early Osteoblast Lineage on the Mineralization of an In Vitro 3D Osteoid-like Dense Collagen Gel Matrix
Source: Biomimetics (Basel). 2022 Jul 22;7(3):101. doi: 10.3390/biomimetics7030101 (PMC9329857; doi:10.3390/biomimetics7030101)
Supplement: Supplementary file 1 [file biomimetics-07-00101-s001.zip › biomimetics-1714207-supplementary.pdf]

Supplemental data

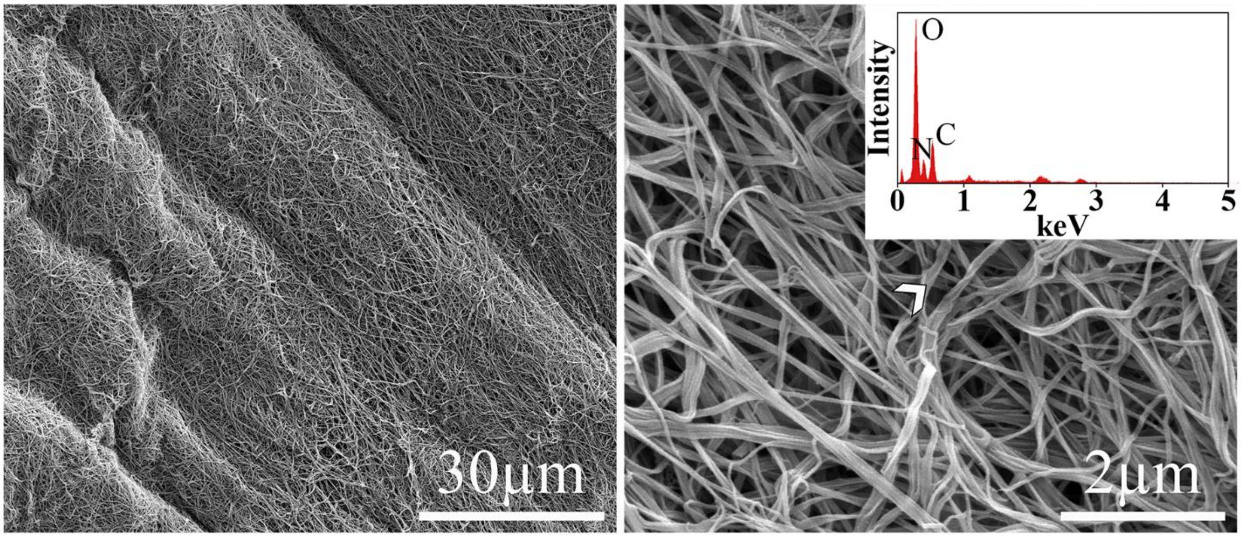

Figure S1. Acellular dense collagen gel at day 21 in osteogenic differentiation medium. Low (left) and high (right) magnification SEM micrographs indicating no mineral deposition. EDS (inset in right panel) on collagen fibril confirmed only the presence of oxygen, nitrogen and carbon and with no calcium or phosphorous.
